# Supplementary material for: Reconciling Southern Ocean fronts equatorward migration with minor Antarctic ice volume change during Miocene cooling
Source: Nat Commun. 2023 Nov 9;14:7230. doi: 10.1038/s41467-023-43106-4 (PMC10636158; doi:10.1038/s41467-023-43106-4)
Supplement: Supplementary file 1 — Supplementary Information [file 41467_2023_43106_MOESM1_ESM.pdf]

Supplementary information for:

Reconciling Southern Ocean fronts equatorward migration with minor Antarctic ice volume change during Miocene cooling

Suning Hou[1]*, Lennert B. Stap[2], Ryan Paul[1], Mei Nelissen[3], Frida S. Hoem[1], Martin Ziegler[1], Appy Sluijs[1], Francesca Sangiorgi[1], Peter K. Bijl[1]

[1]Department of Earth Sciences, Utrecht University, the Netherlands

[2]Institute for Marine and Atmospheric research Utrecht, Utrecht University, the Netherlands

[3]NIOZ Royal Netherlands Institute of Sea Research, Texel, The Netherlands

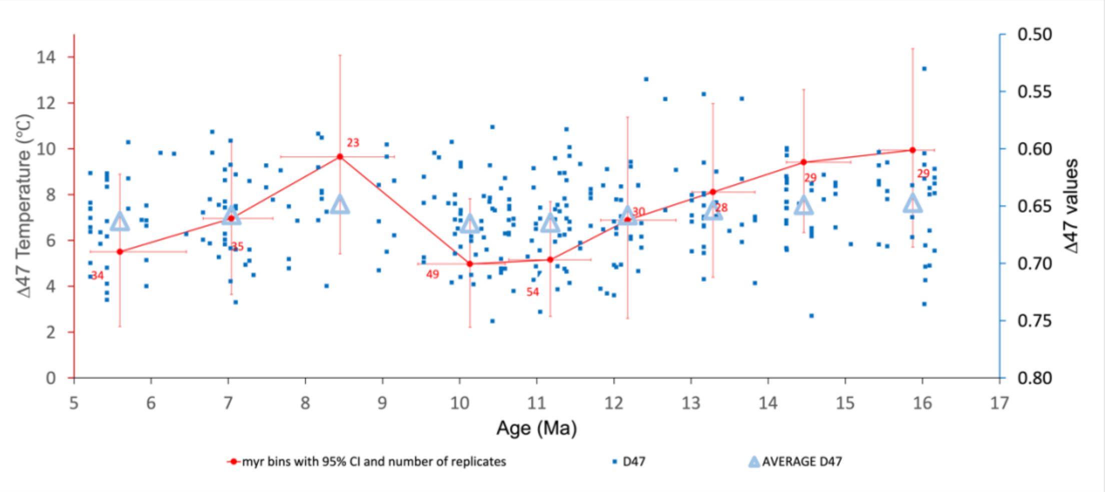

Figure S1: Δ_{47} values and Δ_{47}-temperature. Results of bottom water temperature in million-year bins (red dots, error bar=95% confidence interval) on the main axis; individual Δ_{47} values (dark blue squares) and average Δ_{47} values of million-year bins (light blue triangles) on the secondary y-axis. Horizontal error bars indicate the time interval of each bin. Vertical error bars indicate 95% confidence interval.

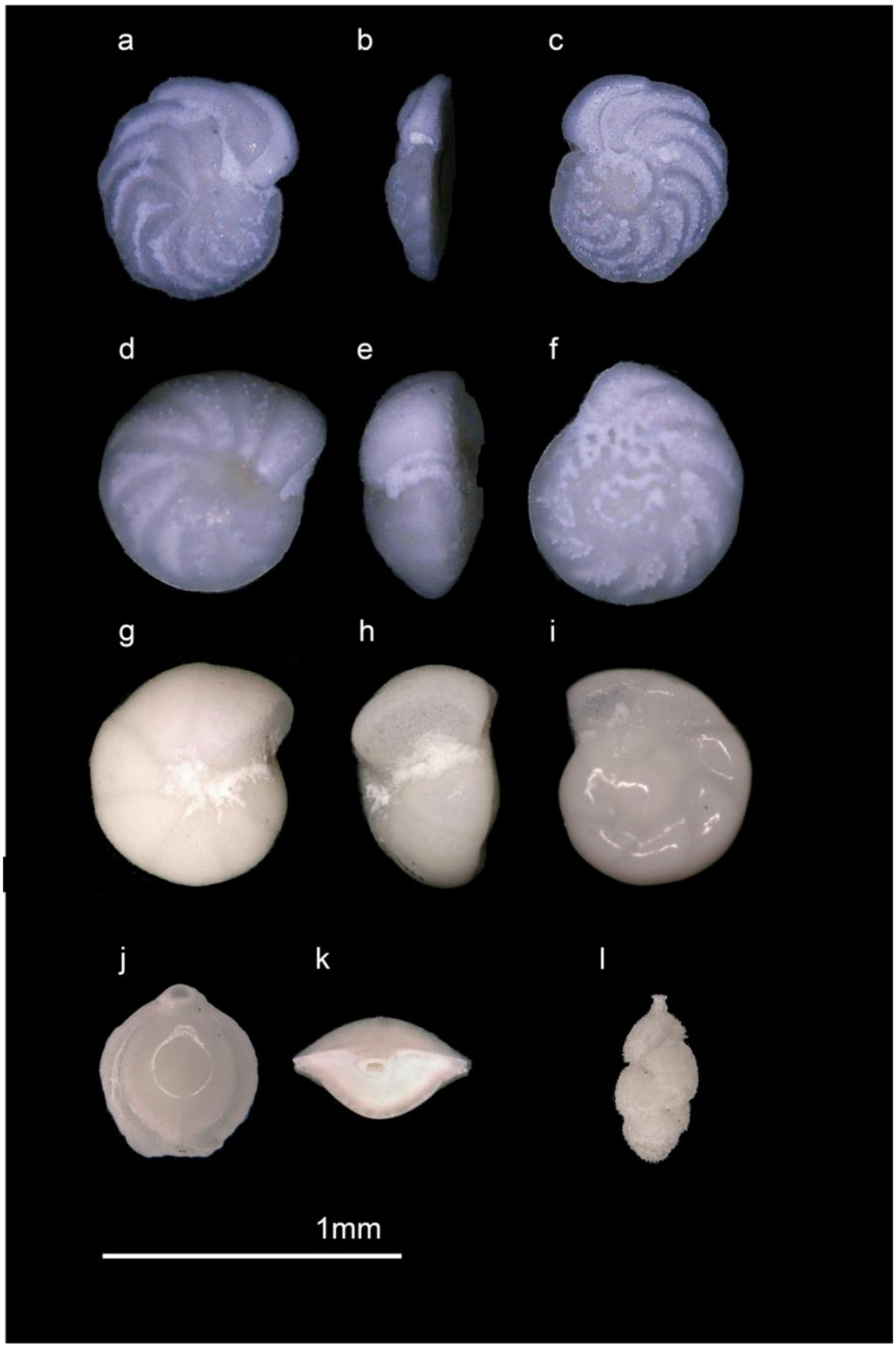

Figure S2: Foraminifera plates of Site 1168. a, b, c : *Cibicidoides wuellerstorffi*; d, e, f: *Cibicidoides mundulus*; g, h, i: *Gyroidina soldanii*; left to right, spiral, aperture and umbilicus views respectively; j, k *Pyrgo* sp.; l: *Uvigerina peregerina.* Foraminiferal specimens were picked from the 250–355 μm size fractions but bot ultrasonically cleaned.

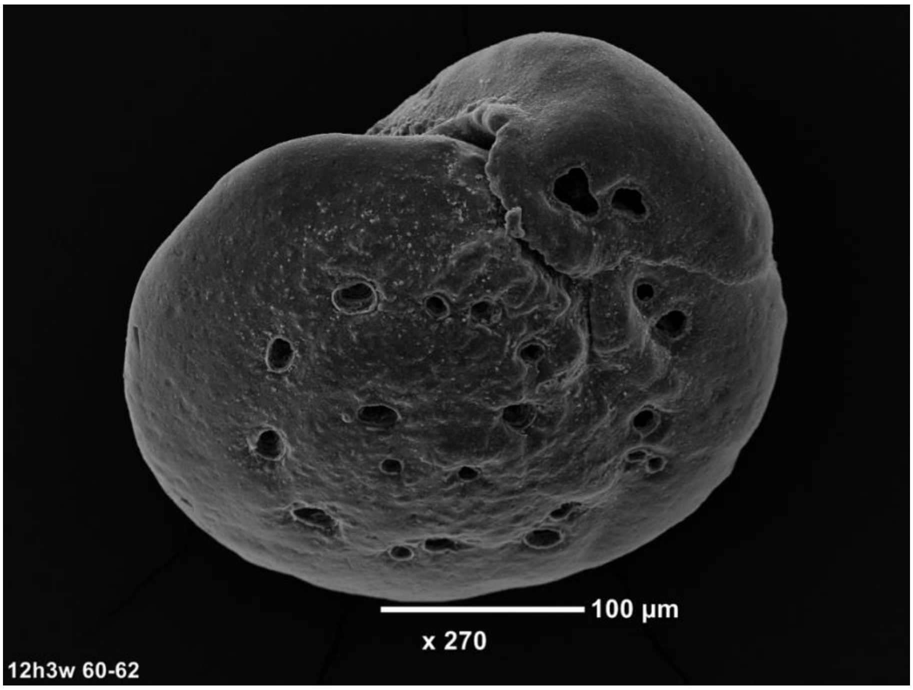

Figure S3: SEM photo of *C. mundulus* of Site 1168. Specimen was obtained from sample 1168A-12H3W, 60-62cm interval, showing the typical state of preservation at Site 1168.

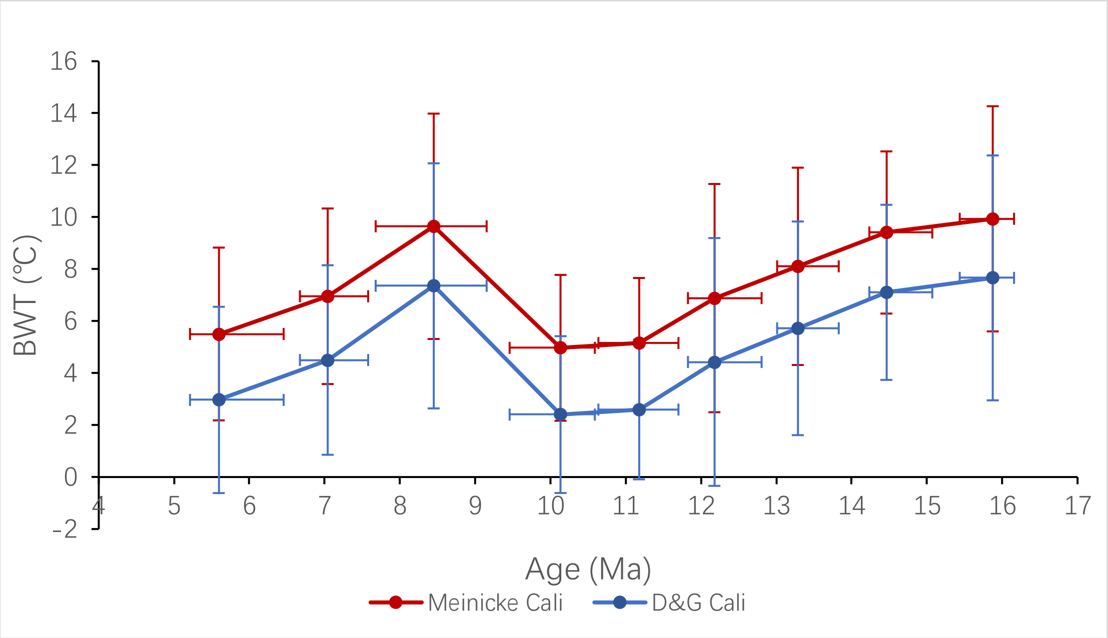

Figure S4: Bottom water temperature using different calibrations. Red line=Meinicke (2020) calibration[1] as in Fig. S1; blue line=Daëron and Gray (2023) calibration[2]. Horizontal error bars indicate the time interval of each bin. Vertical error bars indicate 95% confidence interval.

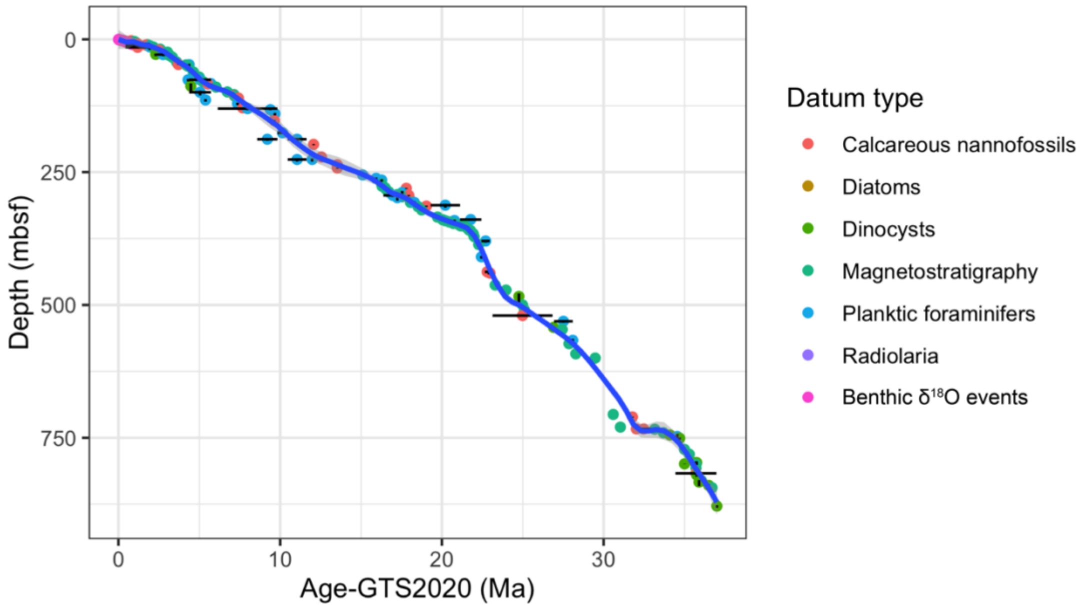

Figure S5: Age model of Site 1168[3,4]. Points indicate the datums. Colors indicate event types. Blue line is the loess smooth curve with a span of 0.1 throughout the studied interval, which we resampled to obtain ages for the samples used in this study. Horizontal error bars indicate the age error. Vertical error bars indicate depth range. Attribution: https://cp.copernicus.org/articles/19/787/2023/ under https://creativecommons.org/licenses/by/4.0/; no changes made.

Table S1: Dinoflagellate cyst groups and ecological/frontal system affinity.

Complex	Genus/species	Ecological affinities/	References
Transported	*Lingulodinium, Homotryblium, Dapsilidinium, Polysphaeridinium*	Inner neritic	Prebble et al.[5]
Spiniferites	*Spiniferites, Achomosphaera, Hafniasphaera, Hystrichostrogylon, Rottnestia*	North of the STF, warm water, Spin-cluster	Thöle et al.[6]
Other Gonyaulacoid	*Acanthaulax, Apteodinium, Ataxiodinium, Batiacasphaera, Caligodinium, Cleistophaeridinium (Systematophora), Dalella,*		

	Hemiplacophora semilunifera, Invertocysta lacrymosa, Labyrinthodinium truncatum, Pentadinium, Pentasphoradium, Reticulatosphaera actinocoronata Stoveracysta, Thalassiphora Tuberculodinium, Turbiosphaera, Unipontidinium, Vozzhennikovia, Other *Operculodinium*		
Other *Impagidinium*	*I. patulum, I. paradoxum, I. dispertum, I. sphaericum, I. pallidum, I. velorum*	Open ocean	Prebble et al.[5]
Nematosphaeropsis	*Nematosphaeropsis*	South of the STF, Nlab-cluster	Thöle et al.[6]
Impagidinium aculeatum	*Impagidinium aculeatum*	South of the STF	Thöle et al.[6]
Operculodinium centrocarpum	*Operculodinium centrocarpum*	North of the STF, High Ocen-cluster	Thöle et al.[6]
Pyxidinopsis	*Filisphaera, Habibacysta, Pyxidinopsis, Tectatodinium, Bitectatodinium, Cerebrocysta*	Cool tolerant	De schepper et al.[7]
Protoperdinium	*Brigantedinium, Echinidinium, Selenopemphix*	High productivity	Thöle et al.[6]

Table S2: Student t-test (two-sample assuming unequal variances) of MCO and 9 Ma Δ47 average values

	Late Miocene (9.5-11 Ma)	MCO (16.5–15.5 Ma)
Mean	0.66520146	0.64861022
Variance	0.00132434	0.0014167
Observations	96	40
Hypothesized Mean Difference	0	
df	71	
t Stat	2.36504657	

P (T<=t) one-tail	0.01038193	
t critical one-tail	1.66659966	
P (T<=t) two-tail	0.02076387	
t critical two-tail	1.99394337	

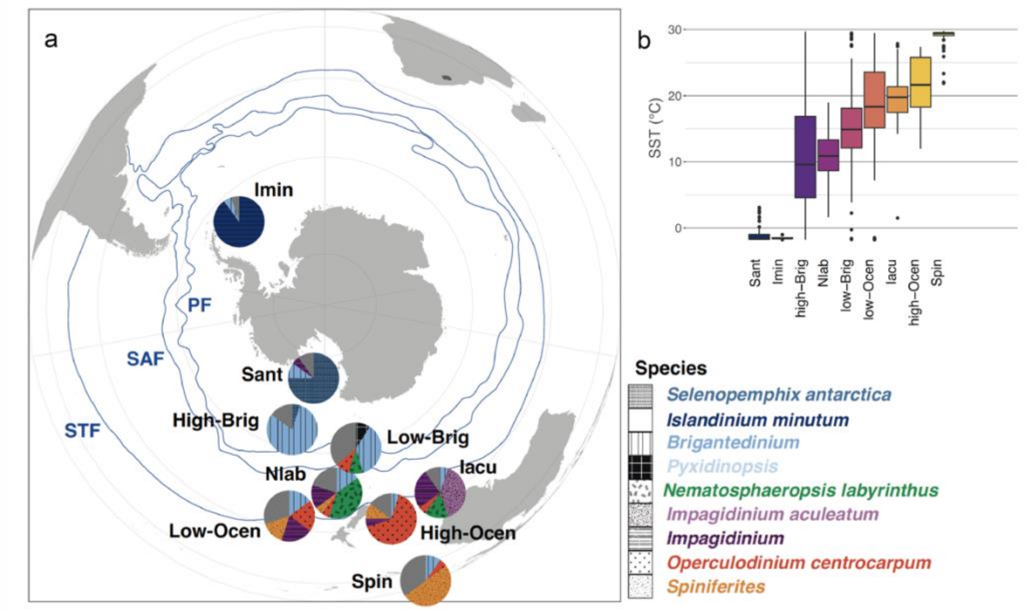

Figure S6: Modern dinocyst clusters distribution in the Southern Ocean. (a). Schematic representation of the generalized biogeographic distribution of dinocysts in Southern Ocean surface sediments. Pies represent average assemblage composition of the nine clusters described in this paper. Position of these pies represent their typical latitudinal band of occurrence. Also plotted are the frontal systems (blue lines, STF = Subtropical Front, SAF = Subantarctic Front, PF = Polar Front). The Subantarctic Zone (SAZ) is the water mass between the STF and PF. (b) Comparison of SST in different clusters for the 9-cluster solution of the sh_655 data set. The median, 25% – 75% quantiles and 95% confidence interval are indicated by the black line, boxes and whiskers, respectively. Modified from Thöle et al.[6].

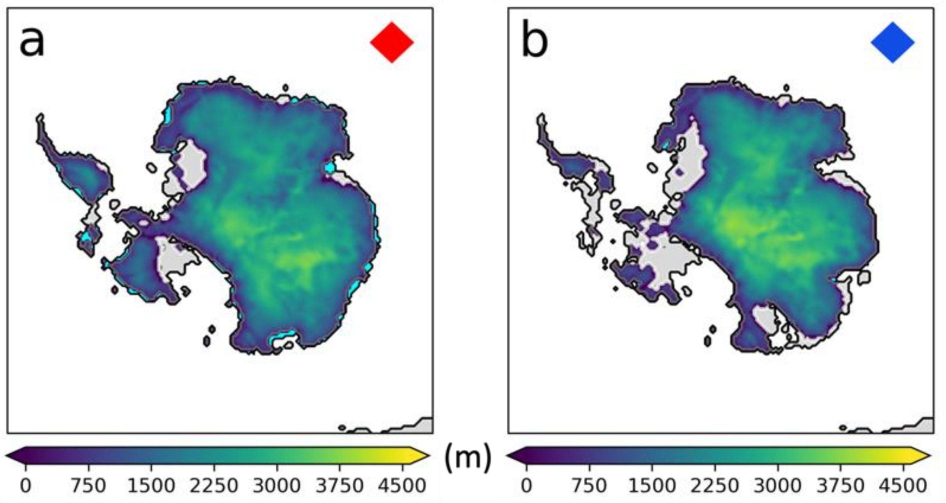

Figure S7: Antarctic ice sheet thickness modelling. (a) Equilibrated ice thickness in the reference simulation at 392 ppm, and (b) in the simulation with anomalous forcing at 504ppm. Grey areas indicate ice-free land, white indicates ocean. Simultaneously reducing the CO_2 level from 504 to 394 ppm and removing the anomalous forcing, leads to significantly larger ice sheet area, while the interior ice sheet height is severely reduced.

Supplementary References:

1. Meinicke, N. *et al.* A robust calibration of the clumped isotopes to temperature relationship for foraminifers. *Geochimica et Cosmochimica Acta* **270**, 160–183 (2020).
2. Daëron, M. & Gray, W. R. Revisiting oxygen-18 and clumped isotopes in planktic and benthic foraminifera. *Paleoceanography and Paleoclimatology* **n/a**, e2023PA004660 (2023).
3. Hou, S. *et al.* Lipid-biomarker-based sea surface temperature record offshore Tasmania over the last 23 million years. *Climate of the Past* **19**, 787–802 (2023).
4. Stickley, C. E. *et al. Proceedings of the Ocean Drilling Program, 189 Scientific Results*. vol. 189 (Ocean Drilling Program, 2004).
5. Prebble, J. G. *et al.* An expanded modern dinoflagellate cyst dataset for the Southwest Pacific and Southern Hemisphere with environmental associations. *Marine Micropaleontology* **101**, 33–48 (2013).
6. Thöle, L. M. *et al.* An expanded database of Southern Hemisphere surface sediment dinoflagellate cyst assemblages and their oceanographic affinities. *Journal of Micropalaeontology* **42**, 35–56 (2023).
7. De Schepper, S., Fischer, E. I., Groeneveld, J., Head, M. J. & Matthiessen, J. Deciphering the palaeoecology of Late Pliocene and Early Pleistocene dinoflagellate cysts. *Palaeogeography, Palaeoclimatology, Palaeoecology* **309**, 17–32 (2011).
