# Supplementary material for: Reconciling Southern Ocean fronts equatorward migration with minor Antarctic ice volume change during Miocene cooling
Source: Nat Commun. 2023 Nov 9;14:7230. doi: 10.1038/s41467-023-43106-4 (PMC10636158; doi:10.1038/s41467-023-43106-4)
Supplement: Supplementary file 3 — Reporting Summary [file 41467_2023_43106_MOESM3_ESM.pdf]

nature portfolio

Corresponding author(s): Suning Hou

Last updated by author(s): Oct 19, 2023

Reporting Summary

Nature Portfolio wishes to improve the reproducibility of the work that we publish. This form provides structure for consistency and transparency in reporting. For further information on Nature Portfolio policies, see our Editorial Policies and the Editorial Policy Checklist.

Statistics

For all statistical analyses, confirm that the following items are present in the figure legend, table legend, main text, or Methods section.

n/a	Confirmed	
☐	☒	The exact sample size (n) for each experimental group/condition, given as a discrete number and unit of measurement
☐	☒	A statement on whether measurements were taken from distinct samples or whether the same sample was measured repeatedly
☐	☒	The statistical test(s) used AND whether they are one- or two-sided<br>*Only common tests should be described solely by name; describe more complex techniques in the Methods section.*
☐	☒	A description of all covariates tested
☒	☐	A description of any assumptions or corrections, such as tests of normality and adjustment for multiple comparisons
☐	☒	A full description of the statistical parameters including central tendency (e.g. means) or other basic estimates (e.g. regression coefficient) AND variation (e.g. standard deviation) or associated estimates of uncertainty (e.g. confidence intervals)
☒	☐	For null hypothesis testing, the test statistic (e.g. F, t, r) with confidence intervals, effect sizes, degrees of freedom and P value noted<br>*Give P values as exact values whenever suitable.*
☒	☐	For Bayesian analysis, information on the choice of priors and Markov chain Monte Carlo settings
☒	☐	For hierarchical and complex designs, identification of the appropriate level for tests and full reporting of outcomes
☒	☐	Estimates of effect sizes (e.g. Cohen's d, Pearson's r), indicating how they were calculated

Our web collection on statistics for biologists contains articles on many of the points above.

Software and code

Policy information about availability of computer code

Data collection	The code for IMAU-ICE v1.1.1-MIO is available from https://github.com/IMAU-paleo/IMAU-ICE/releases/tag/v1.1.1-MIO (last access: 1 September 2023) and https://doi.org/10.5281/zenodo.6352125. The clumpedcalib R package is available from https://github.com/japhir/clumpedcalib.
Data analysis	Not applicable

For manuscripts utilizing custom algorithms or software that are central to the research but not yet described in published literature, software must be made available to editors and reviewers. We strongly encourage code deposition in a community repository (e.g. GitHub). See the Nature Portfolio guidelines for submitting code & software for further information.

Data

Policy information about availability of data

All manuscripts must include a data availability statement. This statement should provide the following information, where applicable:

- Accession codes, unique identifiers, or web links for publicly available datasets
- A description of any restrictions on data availability
- For clinical datasets or third party data, please ensure that the statement adheres to our policy

Raw palynological counting, grouped dinocyst data, dinocyst-based SST, BWT bins and stable isotopes data are archived in Zenodo: https://doi.org/10.5281/zenodo.8146850. Clumped isotope data is archived in the EarthChem database: https://doi.org/10.26022/IEDA/112993. The reference simulations analysed in this

study are openly accessible from the PANGAEA database: https://doi.org/10.1594/PANGAEA.939114. The additional simulations with increased precipitation and sub-shelf melt are available from the Zenodo database: https://doi.org/10.5281/zenodo.8308286.

Research involving human participants, their data, or biological material

Policy information about studies with human participants or human data. See also policy information about sex, gender (identity/presentation), and sexual orientation and race, ethnicity and racism.

Reporting on sex and gender	Not applicable
Reporting on race, ethnicity, or other socially relevant groupings	Not applicable
Population characteristics	Not applicable
Recruitment	Not applicable
Ethics oversight	Not applicable

Note that full information on the approval of the study protocol must also be provided in the manuscript.

Field-specific reporting

Please select the one below that is the best fit for your research. If you are not sure, read the appropriate sections before making your selection.

☐ Life sciences ☐ Behavioural & social sciences ☒ Ecological, evolutionary & environmental sciences

For a reference copy of the document with all sections, see nature.com/documents/nr-reporting-summary-flat.pdf

Ecological, evolutionary & environmental sciences study design

All studies must disclose on these points even when the disclosure is negative.

Study description	We generate for the first time a comprehensive reconstruction of latitudinal migrations of the Southern Ocean subtropical front (STF) in the Neogene (23.0–2.5 Ma), in comparison to ice-proximal and equatorial climate changes. We demonstrate a mid-late Miocene (16–9 Ma) equatorward migration (from ~53°S to ~42°S) and strengthening of the STF, concurrent with SST and BWT drops. The equatorward migration of the STF led to profound deep and surface ocean cooling resulting from a seaward expanded AIS, more abundant ice-ocean interactions and northwards extension of the seasonal sea ice cover. We directly couple this to deep-sea temperature and Antarctic ice sheet dynamics, by presenting new clumped isotope data straddling almost the entire mid-to-late Miocene, filling a critical gap in existing clumped isotope data. We demonstrate that ice sheet models can reproduce such a geometric change, although this would require rigorous interventions into the physics of ice-climate-ocean interactions, such as a warm ocean surrounding Antarctica and increased precipitation.
Research sample	Sediment core from Ocean Drilling Program Leg 189 Site 1168, offshore west Tasmania. This core well recorded the migration of the subtropical front.
Sampling strategy	We basically chose 1 sample (2cm) per section in the core, and some of the samples were already collected in 2004.
Data collection	PKB designed the research. SH, MN and FSH processed and analysed samples for palynology. SH, RP and AS generated the stable isotopes data. SH and RP washed the foraminifera samples and generated the clumped isotope data. LBS performed the ice sheet modelling.
Timing and spatial scale	Data generating started from 2019, ended in 2021.
Data exclusions	Palynological data with less than 50 specimens are discarded.
Reproducibility	Palynology slides can be re-counted.
Randomization	Not applicable
Blinding	Not applicable

Did the study involve field work? ☐ Yes ☒ No

Reporting for specific materials, systems and methods

We require information from authors about some types of materials, experimental systems and methods used in many studies. Here, indicate whether each material, system or method listed is relevant to your study. If you are not sure if a list item applies to your research, read the appropriate section before selecting a response.

Materials & experimental systems

n/a	Involved in the study
☒	☐ Antibodies
☒	☐ Eukaryotic cell lines
☐	☒ Palaeontology and archaeology
☒	☐ Animals and other organisms
☒	☐ Clinical data
☒	☐ Dual use research of concern
☒	☐ Plants

Methods

n/a	Involved in the study
☒	☐ ChIP-seq
☒	☐ Flow cytometry
☒	☐ MRI-based neuroimaging

Palaeontology and Archaeology

Specimen provenance	International Ocean Discovery Program, kochi core repository
Specimen deposition	All materials, including foraminifera and palynology slides are stored in Utrecht University, the Netherlands.
Dating methods	The age model is based on Stickley et al.(2004), revised in Hou et al. (2023).

☒ Tick this box to confirm that the raw and calibrated dates are available in the paper or in Supplementary Information.

Ethics oversight	not applicable

Note that full information on the approval of the study protocol must also be provided in the manuscript.
