# Supplementary material for: Reconciling Southern Ocean fronts equatorward migration with minor Antarctic ice volume change during Miocene cooling
Source: Nat Commun. 2023 Nov 9;14:7230. doi: 10.1038/s41467-023-43106-4 (PMC10636158; doi:10.1038/s41467-023-43106-4)
Supplement: Supplementary file 4 — Description of Additional Supplementary Files [file 41467_2023_43106_MOESM4_ESM.pdf]

Description of Additional Supplementary Files

File Name: Supplementary Data 1
Description: Clumped isotope data averages with confidence interval, temperatures and $\delta^{18}O_{sw}$ values
